# A Randomized Placebo-Controlled Trial of Acetaminophen for Prevention of Post-Vaccination Fever in Infants

**DOI:** 10.1371/journal.pone.0020102

**Published:** 2011-06-17

**Authors:** Lisa A. Jackson, Do Peterson, John Dunn, Simon J. Hambidge, Maya Dunstan, Patty Starkovich, Onchee Yu, Joyce Benoit, Clara P. Dominguez-Islas, Barbara Carste, Patti Benson, Jennifer C. Nelson

**Affiliations:** 1 Group Health Research Institute, Seattle, Washington, United States of America; 2 Institute for Health Research, Kaiser Permanente Colorado and Denver Health Community Health Services, Denver, Colorado, United States of America; 3 Department of Biostatistics, University of Washington, Seattle, Washington, United States of America; Genentech Inc., United States of America

## Abstract

**Background:**

Fever is common following infant vaccinations. Two randomized controlled trials demonstrated the efficacy of acetaminophen prophylaxis in preventing fever after whole cell pertussis vaccination, but acetaminophen prophylaxis has not been evaluated for prevention of fever following contemporary vaccines recommended for infants in the United States.

**Methods:**

Children six weeks through nine months of age were randomized 1∶1 to receive up to five doses of acetaminophen (10–15 mg per kg) or placebo following routine vaccinations. The primary outcome was a rectal temperature ≥38°C within 32 hours following the vaccinations. Secondary outcomes included medical utilization, infant fussiness, and parents' time lost from work. Parents could request unblinding of the treatment assignment if the child developed fever or symptoms that would warrant supplementary acetaminophen treatment for children who had been receiving placebo.

**Results:**

A temperature ≥38°C was recorded for 14% (25/176) of children randomized to acetaminophen compared with 22% (37/176) of those randomized to placebo but that difference was not statistically significant (relative risk [RR], 0.63; 95% CI, 0.40–1.01). Children randomized to acetaminophen were less likely to be reported as being much more fussy than usual (10% vs 24%) (RR, 0.42; 95% CI, 0.25–0.70) or to have the treatment assignment unblinded (3% vs 9%) (RR, 0.31; 95% CI, 0.11–0.83) than those randomized to placebo. In age-stratified analyses, among children ≥24 weeks of age, there was a significantly lower risk of temperature ≥38°C in the acetaminophen group (13% vs. 25%; p = 0.03).

**Conclusion:**

The results of this relatively small trial suggest that acetaminophen may reduce the risk of post-vaccination fever and fussiness.

**Trial registration:**

Clinicaltrials.gov NCT00325819

## Introduction

Fever is a relatively common adverse event following administration of vaccines routinely recommended for infants under one year of age. For example, a randomized trial of seven-valent pneumococcal conjugate vaccine given concomitantly with diphtheria and tetanus toxoid and acellular pertussis (DTaP) and other routinely recommended vaccines at two, four, and six months of age reported that up to 24% of participants had a rectal temperature ≥38°C and up to 2.5% had a rectal temperature ≥39°C within 48 hours of vaccination.[1] Another trial of pneumococcal conjugate vaccine given with a combination DTaP, hepatitis B, inactivated poliovirus, and *Haemophilus influenzae* type b (DTaP-HepB-IPV-Hib) vaccine at two, three, and four months of age reported that up to 49% of participants had a rectal temperature ≥38°C and up to 4.6% had a rectal temperature ≥39°C within four days of vaccination.[2] Although significant adverse events, such as febrile seizures, can occur, even in the acellular pertussis vaccine era,[3], [4] these events are infrequent and post-vaccination fever in young infants is generally self-limited. Post-vaccination fever can, however, lead to emergency room visits and other medical utilization and can cause a parent to miss time from work to care for the febrile child. Fever also results in discomfort for the child and may lead to disruption of sleep for both the parents and the child. Lastly, the occurrence of post-vaccination fever could potentially influence parents' perception of the safety of routine childhood immunizations.

Two randomized controlled trials conducted in the 1980s reported reductions in risk of post-vaccination fever in infants two through six months of age who received acetaminophen at the time of whole cell pertussis vaccination, which was associated with significant reactogenicity.[5], [6] In those trials, acetaminophen prophylaxis also led to reductions in the proportion of infants with fussiness and persistent crying in the day following vaccination. Prophylactic use of acetaminophen has not been evaluated in infants receiving contemporary vaccines routinely recommended for use in the United States.

We designed this randomized, placebo controlled trial to evaluate the possible benefits of acetaminophen prophylaxis for the prevention of post-vaccination fever and other outcomes, including medical utilization and parents' time lost from work, among infants less than ten months of age receiving routinely recommended vaccinations. The study sample size of 1000 children was selected to have 80% power to detect a 30% reduction in risk of the primary outcome of rectal temperature ≥38°C following vaccination. In 2009, during the enrollment period of our trial, Prymula and colleagues reported the results of a randomized trial of acetaminophen prophylaxis in infants in the Czech Republic receiving a primary series of ten-valent pneumococcal non-typeable *Haemophilus influenzae* protein D-conjugate vaccine (PHiD-CV) co-administered with a combination DTaP-HepB-IPV-Hib vaccine and oral human rotavirus vaccine.[7] The trial included evaluations of immunogenicity and found significantly lower immune responses to all ten pneumococcal vaccine serotypes and to Hib polysaccharide, diphtheria, tetanus, and pertactin antigens in the acetaminophen group. In light of these unexpected findings indicating a detrimental effect of acetaminophen prophylaxis on vaccine immune response, which could at least theoretically have clinical implications, we elected to stop enrollment in our trial. Here we report evaluation of the 352 children enrolled prior to study cessation.

## Methods

The protocol for this trial and supporting CONSORT checklist are available as supporting information; see Checklist S1 and Protocol S1.

### Ethics statement

The study was conducted in compliance with the Helsinki Declaration and was approved by the Institutional Review Boards at Group Health and the Centers for Disease Control and Prevention.

### Study design and population

We conducted a randomized, observer and participant blinded, placebo controlled trial of acetaminophen prophylaxis among children less than 10 months of age enrolled in Group Health Cooperative, a managed care organization in Washington State. Children were eligible for enrollment if they were expected to receive two or more injected vaccines at an upcoming well child visit occurring after six weeks and before 10 months of age. Children less than four months of age who had a birth weight of <2500 grams or gestational age of <36 weeks were excluded from enrollment but children four through nine months of age could be enrolled regardless of birth weight or gestational age.

### Recruitment, enrollment, and randomization

Children potentially eligible for study participation were identified from the Group Health data systems and parents were mailed a letter providing information on the study and inviting them to contact the study team if they were interested in more information. If the child was confirmed to be eligible by phone interview with the parents and by medical record review, a consent form was mailed to the parents for them to sign and return.

After the parents returned the signed consent form, the child was randomized with equal probability to receive acetaminophen or placebo. The randomization sequence was generated by the study biostatistician and provided to the study pharmacy. To ensure balanced allocation, the randomization schedule was generated with a variable block size of between six and twelve. Neither the study biostatistician nor the study pharmacist was involved with the enrollment of participants.

At a time proximate to the scheduled well child vaccination visit, the parents were mailed an enrollment package that included the bottle of study medication, two 3.0 mL medication syringes graduated in 0.5 mL intervals, a digital thermometer, the study diary, and the study medication dosing instructions.

### Study drug, dosage, and timing

The liquid placebo and the acetaminophen suspension were flavored to mask differences in taste and packaged by the study pharmacy in identical containers identified only by study id number. The acetaminophen suspension was formulated to provide 160 mg of acetaminophen per 5 mL dose volume. The parents were instructed to use the dosage table provided in the enrollment package to identify the recommended dose volume based on the child's weight, which would provide between 10 mg and 15 mg of acetaminophen per kilogram. Parents and study staff members involved in recruitment, enrollment, and follow up were blinded to study assignment.

Parents were encouraged to take the bottle of study medication to the vaccination visit, and to give the first dose at that visit and within an hour before or after the vaccinations. If the parent was unable to give the first dose within an hour of vaccination, they were asked to give the first dose as close as possible to the vaccination, and within the allowable window of four hours before through up to 24 hours after the vaccinations.

Following the initial dose, parents were instructed to give subsequent doses no earlier than, but as close to, four hours following the previous dose as possible. A maximum of five doses of study medication should be given, and the last dose must be given no later than 24 hours after the study vaccination regardless of the total number of doses administered. If all doses were given exactly on schedule, doses would be given at 0, 4, 8, 12, and 16 hours after vaccination.

### Data collection

Parents completed a study diary and recorded the child's weight (measured at the vaccination visit), the time that vaccinations were administered, and the timing and volume of each dose of study medication administered. If they discontinued the study medication, they were asked to record the reason(s).

Parents were asked to take the child's rectal temperature just prior to administration of the second, third, fourth, and fifth doses of study medication and to take a final rectal temperature approximately 24 hours after vaccination, or four hours after the final dose of study medication, whichever was later. At each study medication dosage time, and at the time of the final temperature assessment, parents were also asked to record the child's level of fussiness.

Parents were also asked to record the relative amount of sleep that each parent and the child had on the night following the vaccinations and were asked to report whether they were scheduled to work on the day of or the day following the child's vaccination visit and, if so, whether either parent missed work to care for the child due to fever, fussiness, or possible vaccine reaction. Parents were to record any use of medical services for fever or other acute symptoms following the vaccination visit through the next day. To judge the adequacy of the blinding procedure, parents were also asked to indicate their guess as to whether their child received acetaminophen or placebo.

Vaccinations given at the vaccination visit were identified from Group Health immunization records.

### Provisions for unblinding

The parents were instructed that if they or a health care provider believed the child needed acetaminophen treatment for any reason, the parent or health care provider could call study staff at any time to request unblinding of the randomization assignment. Study staff could unblind a child's assignment by opening an individual, sealed envelope labeled with the child's study number. After unblinding, study staff referred the parent to the consulting nurse or the child's physician for further clinical management, if needed, of the febrile illness or other symptoms that led to the request for unblinding.

### Primary and secondary outcomes

The primary outcome was a rectal temperature ≥38°C within 32 hours of vaccination. This time interval was chosen because it corresponds to 8 hours after the latest possible administration of the last dose of study medication (to be given no later than 24 hours after vaccination) and thus encompasses the maximum window of expected activity of the study medication.

Secondary outcomes and their definitions are as follows. All outcomes were specified a priori

Rectal temperature ≥39°C within 32 hours of vaccination.

Medical utilization. Telephone calls to the consulting nurse or the child's physician that were made due to concerns regarding an acute illness, fever, or possible vaccine reaction and outpatient, urgent care, and emergency room visits that were for evaluation of an acute illness, fever, or a possible vaccine reaction, within 32 hours of vaccination.

Parent time lost from work. Parents were asked to report whether they were scheduled to work on the day of the vaccination visit, but following that visit, or the next day and, if so, whether they had to miss work to care for their infant because of fever, fussiness, or possible vaccine reaction on those days.

Time lost from sleep. Parents were asked about their sleep and the child's sleep on the night following the vaccinations. They were asked to report whether they and the infant slept much less than usual, less than usual, about the usual amount, more than usual, or much more than usual on that night.

Infant fussiness. Parents were asked to record level of fussiness (compared with the child's usual) within 32 hours of vaccination, using the categories much less than usual, less than usual, about usual, more than usual, and much more than usual.

Unblinding of study drug assignment. The need for unblinding, including the timing of and the precipitating reason, was assessed.

## Sample size

Assuming that 40% of children in the placebo group would have the primary outcome of temperature ≥38°C, a sample size of 897 would allow 80% power to detect a 30% reduction in risk of that outcome in the treatment group. The sample size was inflated by about 10% to account for failure to return study diaries for a final intended sample size of 1000. As previously mentioned, the study was stopped at about one-third of the intended enrollment and so the sample size achieved did not allow adequate power to identify the pre-specified 30% reduction in risk of the primary outcome.

## Statistical analysis

The primary analysis included all participants who received at least one dose of study drug and had study diary information reported. Due to the bimodal age distribution of the study population, exploratory age-stratified analyses of the age groups <24 weeks and ≥24 weeks were also performed. The per protocol population was defined by administration of the first dose of the study medication within four hours before through one hour after vaccination, and administration of at least two additional doses, given at least 4 hours apart, within 24 hours of vaccination. The results of the per protocol analyses were very similar to those of the intent-to-treat analyses and are not presented.

Descriptive statistics including percentages for binary and categorical variables and means and standard deviations for continuously scored variables were computed by treatment group. Relative risks of primary and secondary outcomes for treated compared to untreated participants were estimated using unadjusted Poisson regression. Robust inference was carried out using empirical Huber-White (sandwich) standard errors because the Poisson model assumption of mean and variance equality did not appear to hold.[8] Statistical analyses were run using SAS 9.2 and STATA 11.0.

## Results

**Figure 1:**
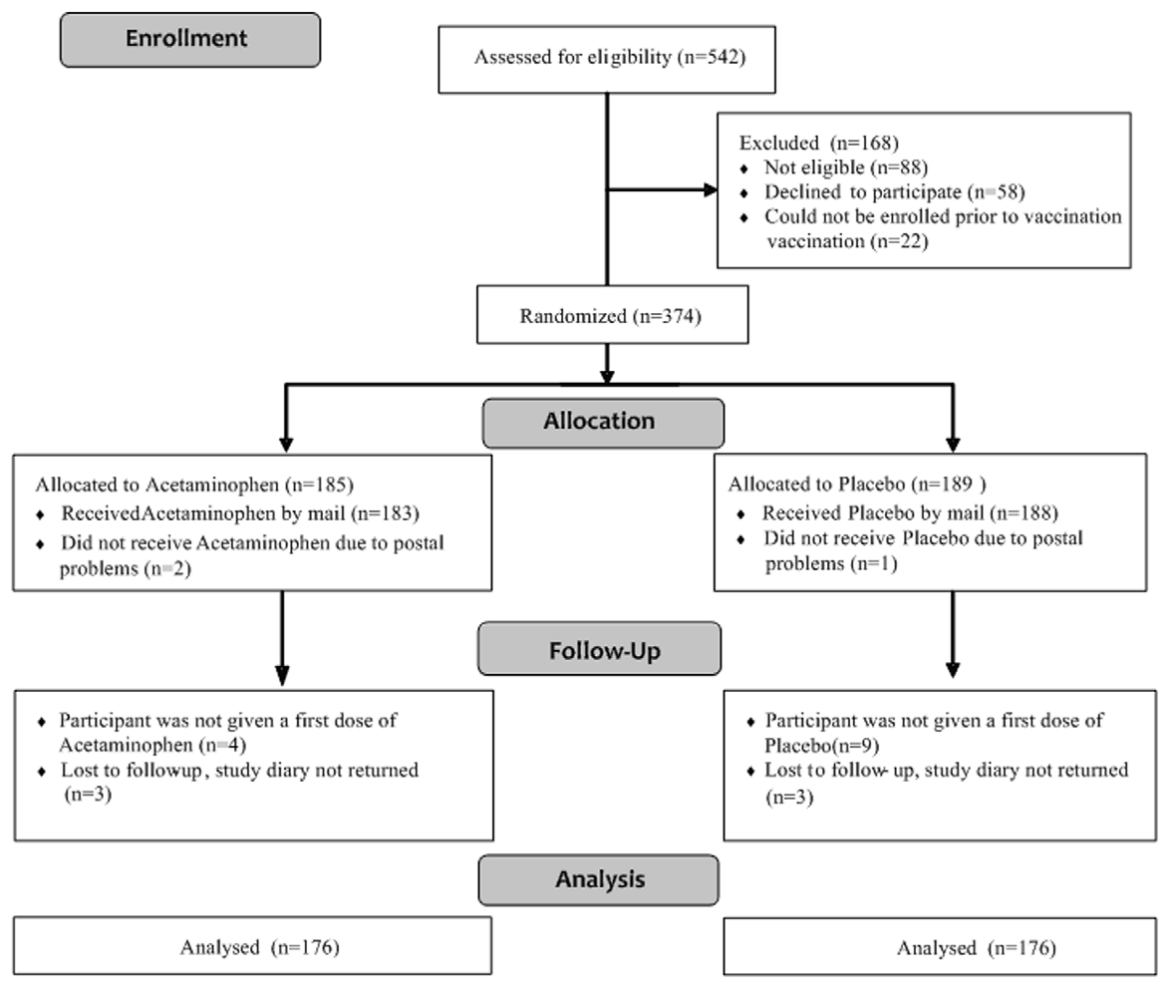
Enrollment Flowchart.

**Figure 2:**
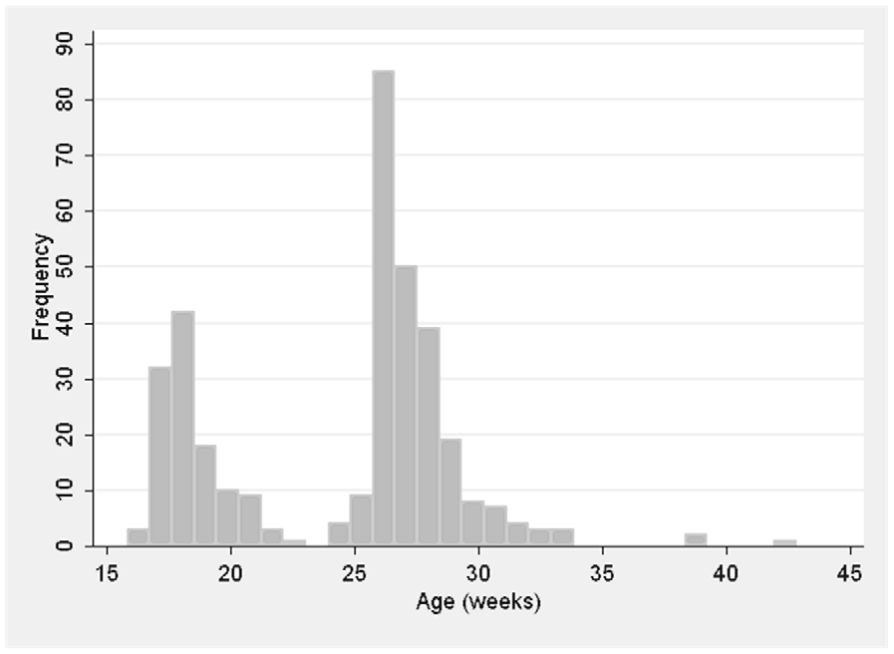
Distribution of participant age in weeks at the vaccination visit.

A total of 374 children were randomized and mailed the study product. Of those, 352, who were vaccinated between June 6, 2006 and September 28, 2009, received at least one dose of study medication and had a completed study diary returned (Figure 1). Those 352 children represent the intent to treat population assessed in the primary study analyses. The age range of those participants was 16 through 42 weeks with a bimodal distribution reflecting ages grouped around the times of the four and six month vaccinations (Figure 2). Other baseline characteristics are shown in Table 1. A subgroup of 234 children (124 acetaminophen and 110 placebo) met the per protocol definition.

**Table 1:** Baseline characteristics and vaccinations received by study group.

		AcetaminophenN = 176	PlaceboN = 176
Age in weeks, mean (SD)		24.8 (4.7)	24.2 (4.7)
Age	16–23 weeks, %	31	36
	24–42 weeks, %	69	64
Female, %		44	54
Weight in lbs, mean (SD)		16.7 (2.3)	16.5 (2.1)
Number of injected vaccines administered, %			
	2	3	3
	3	15	16
	4	69	69
	5	13	11
	6	0	1
Vaccines administered, %			
	DTaP	62	61
	DTaP-HepB-IPV	19	16
	DTaP-IPV/Hib	17	21
	HepB	11	11
	Hib	75	74
	Hib-HepB	2	3
	IPV	60	61
	PCV7	98	98
	TIV	11	10

DTaP, Diphtheria and tetanus toxoids and acellular pertussis vaccine; HepB, hepatitis B vaccine; IPV, inactivated poliovirus vaccine; Hib, Haemophilus influenzae type b conjugate vaccine; PCV7, pneumococcal conjugate vaccine (7-valent); TIV, trivalent inactivated influenza vaccine.

The first dose of study medication was given within one hour of vaccination for 99% of subjects in each treatment group. In the acetaminophen group, 98% received at least 2 doses, 94% at least 3 doses, 78% at least 4 doses, and 33% received 5 doses. In the placebo group, 98% received at least 2 doses, 91% at least 3 doses, 76% at least 4 doses, and 31% received 5 doses.

Children in the acetaminophen group were less likely to have the primary outcome of temperature ≥38°C than children in the placebo group, but this difference was not statistically significant (p = 0.05) (Table 2). In analyses of the secondary outcomes, children randomized to acetaminophen were less likely to be reported as being much more fussy than usual or to have the treatment assignment unblinded than those randomized to placebo but there was no significant difference between the groups for the other secondary outcomes. High fever (temperature ≥39°C) was reported in only three (2%) placebo recipients. Febrile seizure, which was assessed as a safety outcome, was not reported in any participant. Of the 19 subjects with treatment assignment unblinded, fever was indicated as a reason for unblinding for 11. Of the remaining eight, seven were unblinded due to fussiness or screaming and one because the child's pediatrician requested administration of acetaminophen due to history of seizure.

**Table 2:** Primary and secondary outcomes by study group.

	AcetaminophenN = 176	PlaceboN = 176	Relative risk (95% CI)	P value**
	%	%		
*Primary outcome*				
Rectal temperature ≥38°C*	14	22	0.66 (0.41, 1.01)	0.053
*Secondary outcomes*				
Rectal temperature ≥39°C*	0	2	--	0.08^F^
Study assignment unblinded	3	9	0.31 (0.12, 0.84)	0.02
Medical utilization	3	6	0.45 (0.16, 1.28)	0.14
Infant fussiness (maximum recorded)				
About usual or less than usual	42	38	Reference	
More than or much more thanusual	58	62	0.94 (0.79, 1.11)	0.45
Much more than usual	10	24	0.40 (0.25, 0.70)	0.001****
Parent sleep**				
About usual or more than usual	73	77	Reference	
Less than or much less thanusual	27	23	1.20 (0.85, 1.80)	0.33
Much less than usual	3	5	0.62 (0.21, 1.88)	0.40****
Infant sleep				
About usual or more than usual	78	81	Reference	
Less than or much less than usual	22	19	1.15 (0.76, 1.75)	0.51
Much less than usual	2	2	1.00 (0.25, 3.94)	1.00****
Missed work, among parents scheduled to work†	4	1	2.94 (0.60, 14.34)	0.18

F Fisher's exact test.

*Temperature values were missing for one subject in the acetaminophen group and two subjects in the placebo group; the analyses included N = 175 in the acetaminophen group and N = 174 in the placebo group.

**If two parents reported, selected parent with the least amount of sleep.

***P-values from Poisson regression with robust variance unless otherwise indicated.

****All other less extreme Likert categories as reference.

† N = 143 in the acetaminophen group and 140 in the placebo group.

In analyses stratified by age (<24 and ≥24 weeks), among children in the older subgroup, there was a significant reduction in risk of temperature ≥38°C in the acetaminophen compared with the placebo group (13% vs. 25%; p = 0.03) that was not found in the younger age group (16% versus 18%; p = 0.8). In both age groups, children randomized to acetaminophen tended to be less likely than children randomized to placebo to be reported as being much more fussy than usual (≥24 weeks, 9% vs 23%; p = 0.004) (<24 weeks, 13% vs 27%; p = 0.055). Among children in the placebo group, those ≥24 weeks of age were not significantly more likely than the younger group to have a temperature ≥38°C (25% vs 18%; p = 0.31) or to be reported as being much more fussy than usual (23% vs 27%; p = 0.56).

To assess the adequacy of the blinding of the study drug, parents were asked to guess whether their child had received acetaminophen or placebo. Fifty-seven percent of parents of children assigned to acetaminophen and 53% of parents of children assigned to placebo correctly guessed the study drug assignment. These proportions were not different than those expected by chance alone and suggested adequate blinding of the study drug.

## Discussion

In this randomized placebo controlled trial of acetaminophen prophylaxis among children less than 10 months of age, we found suggestions of a benefit of acetaminophen in reducing the risk of fever and increased fussiness following vaccination. Although the risk of the primary outcome of temperature ≥38°C was lower in the acetaminophen compared to the placebo group (15% versus 22%), this difference was not statistically significant in the primary analysis of all subjects. A significantly lower risk of the primary outcome was found in the subgroup analysis of infants ≥24 weeks of age. Among all participants, children randomized to acetaminophen were less likely to be described as much more fussy than usual during the study period and to have had the study assignment unblinded. Temperature ≥39°C was uncommon, and was found in only 3 of 176 (2%) placebo recipients.

These possible benefits are consistent with the findings of two randomized controlled trials of acetaminophen prophylaxis in children receiving whole cell pertussis vaccine, which included primarily children two through six months of age. In the trial by Ipp, the risk of temperature ≥38°C was 39% lower in the acetaminophen group (RR, 0.61; 95% CI, 0.47–0.81), with similar reductions in fussiness/fretfulness and persistent crying.[5] In the trial by Lewis, the risk of temperature ≥38°C was 43% lower in the acetaminophen group (RR, 0.57; 95% CI, 0.41–0.79), with a similar reduction in fussiness/fretfulness.[6] In both of those studies, the risk of fever among placebo recipients was relatively high. A temperature of ≥38°C was found in 43% of placebo recipients in the Ipp study and 53% in the Lewis study and a temperature ≥39°C was found in 13% of placebo recipients in the Ipp study. The prevalence of fever is consistent with the higher reactogenicity of whole cell pertussis vaccine formulations compared with contemporary acellular pertussis vaccine formulations. Unlike whole cell pertussis vaccines, DTaP vaccines do not appear to be associated with a substantially increased risk of post-vaccination febrile seizures.[9]

Our results are also consistent with the results of a recent randomized, open label study of acetaminophen (paracetamol) prophylaxis given with PhiD-CV and DTaP-HBV-IPV/Hib vaccines administered at three, four, and five months of age compared with no prophylaxis.[7] The primary outcome of rectal temperature ≥38°C during the four days after any of those vaccinations was significantly less common in the acetaminophen group (42%) than the comparison group (66%).

In that study, a unexpected detrimental effect of acetaminophen prophylaxis on immune responses to vaccine antigens was found. Although most children who received acetaminophen achieved an immune response believed to correlate with protection, the responses, as measured by ELISA or by an opsonophagocytic assay, to the pneumococcal serotypes included in the vaccine tended to be lower in the acetaminophen group. In addition, there were lower immune responses to Hib polysaccharide, diphtheria, tetanus, and pertactin antigens in the acetaminophen group. Post hoc analyses of previous vaccine clinical trials, with information on concomitant acetaminophen use, also found similar trends for an association of acetaminophen exposure and reduced responses to pneumococcal conjugate vaccine.[7]

Although the clinical relevance of these findings is not known, the study authors, and the authors of the accompanying editorial,[10] interpreted the findings as arguing against routine acetaminophen prophylaxis at the time of vaccination. We agreed that, in the absence of additional information, the potential benefit of acetaminophen prophylaxis in reducing the risk of fever and associated adverse events following contemporary infant immunizations appears to be outweighed by the potential harmful effects of acetaminophen prophylaxis on vaccine immune responses and we stopped enrollment in our trial after the publication of the paper by Prymula and colleagues in October 2009. Subsequent to that time, we used Group Health databases to identify study participants who had been hospitalized for any reason between the time of study enrollment and their second birthday and identified 14 children who had been hospitalized during that period. We evaluated the discharge diagnoses assigned to the 14 hospitalizations and identified none that appeared due to a potentially vaccine preventable infection, such as pneumococcal or Hib infection.

### Conclusions

In summary, the results of this trial that included about one third of the predefined sample size suggest a benefit of acetaminophen prophylaxis in reducing fever among infants receiving DTaP vaccine and other currently recommended vaccines. New information demonstrating an adverse effect of acetaminophen prophylaxis on vaccine immune response, as well as data indicating that the risk of febrile seizures, a more serious complication of post-vaccination fever, is not increased following administration of DTaP vaccine, indicates that acetaminophen prophylaxis should not be routinely used for prevention of post-vaccination fever. Future evaluations of acetaminophen or other anti-inflammatory drugs given in association with vaccinations should include evaluations of vaccine immune response.

The findings and conclusions in this report are those of the authors, and do not necessarily represent the official position of the Centers for Disease Control and Prevention.

## Supporting Information

Checklist S1 CONSORT checklist.
(DOC)

Protocol S1 Protocol for this trial.
(DOC)
