# Supplementary material for: A Randomized Placebo-Controlled Trial of Acetaminophen for Prevention of Post-Vaccination Fever in Infants
Source: PLoS One. 2011 Jun 17;6(6):e20102. doi: 10.1371/journal.pone.0020102 (PMC3117800; doi:10.1371/journal.pone.0020102)
Supplement: Protocol S1 — Protocol for this trial. (DOC) [file pone.0020102.s002.doc]

**A randomized placebo-controlled trial of acetaminophen for prevention of**

# post-vaccination fever in infants

Investigators

Lisa Jackson, MD, MPH

Principal Investigator

Center for Health Studies, Group Health Cooperative

Jennifer Nelson, PhD

Study biostatistician

Center for Health Studies, Group Health Cooperative

John Dunn, MD, MPH

Co-investigator

Department of Pediatrics, Group Health Cooperative

Simon Hambidge, MD, PhD

Co-investigator

Kaiser Colorado

Frank DeStefano, MD, MPH

Co-investigator

Immunization Safety Office, Office of the Chief Science Officer

Centers for Disease Control and Prevention

Medical Monitor

Janet Englund, MD

Associate Professor of Pediatrics

Children’s Regional Medical Center, University of Washington

Table of contents

## SYNOPSIS 3

**A. INTRODUCTION** 4

A.1. Introduction 4

A.2. Role of CDC Investigator 4

**B. BACKGROUND** 4

B.1. Fever following administration of pneumococcal conjugate

and other currently recommended vaccinations 4

B.2. Trials of acetaminophen prophylaxis prior to

whole cell pertussis vaccination 5

B.3. Current recommendations for prophylaxis prior to childhood vaccinations 7

B.4. Significance of the current proposal 7

**C. RESEARCH DESIGN AND METHODS** 7

C.1. Study design 7

C.2. Study population 7

C.3. Inclusion/exclusion criteria 8

C.4. Recruitment and enrollment 8

C.5. Randomization 9

C.6. Study drug 9

C.6.a. Dosage and timing of doses 9

C.6.b. Study drug formulation and dispensement 9

C.7. Provisions for unblinding 10

C.8. Outcomes 11

C.9. Data collection 12

C.10. Power calculations and sample size 13

C.11. Statistical analysis 13

**D. EFFICACY AND SAFETY MONITORING** 13

D.1. Efficacy monitoring 13

D.2. Safety monitoring 14

D.2.a. Safety outcomes 14

### TABLES

Table 1 5

Table 2 5

Table 3 6

Table 4 6

Table 5 9

Table 6 10

**REFERENCE LIST** 16

# Synopsis

# Background. Post-vaccination fever occurs in up to 40% of infants receiving routinely recommended childhood vaccinations. Although serious events are rare, post-vaccination fever causes discomfort for the child, can lead to medical utilization, can rarely result in febrile seizure, and can cause a working parent to miss time from their job to care for a febrile infant who cannot attend day care. Two randomized controlled trials demonstrated the efficacy of acetaminophen prophylaxis in preventing fever after whole cell pertussis vaccination, but acetaminophen prophylaxis has not been evaluated for prevention of fever following acellular pertussis and other currently recommended infant vaccinations. The benefits of acetaminophen prophylaxis for infants receiving current vaccinations, in terms of reduction of discomfort for the child, improvement of quality-of-life indicators for the parent, or reduction of medical utilization, have therefore not been measured.

# Primary Objective. To assess the efficacy of prophylaxis with acetaminophen in prevention of fever following routine childhood immunizations.

**Design**. Randomized, blinded, placebo-controlled trial.

**Setting.** Group Health Cooperative, a large health maintenance organization in Washington State.

**Participants.** 1000 children six weeks through nine months of age.

**Intervention.** Children will be randomly assigned with equal probability to receive either acetaminophen or placebo. Parents will be instructed to give the first dose within one hour before or after vaccination, and to give up to four subsequent doses in the next 24 hours, with a minimum of four hours between consecutive doses.

**Sample size.** Assuming a frequency of the primary outcome event of 40% in the placebo group, evaluation of 897 children would allow 80% power to detect a 30% reduction in risk of the primary outcome associated with treatment. Assuming that 10% of randomized participants will not be evaluable, randomization of 1000 children would give an adequate number of evaluable participants to achieve 80% power to detect a 30% reduction in risk of the primary outcome.

# A. Introduction

# A.1. Introduction

Each month, approximately one million infants in the United States receive vaccinations recommended routinely at 2, 4, and 6 months of age. Up to 40% of those infants will have a temperature ≥38ºC following vaccination.^1^ Although significant adverse events, such as febrile seizures, can occur, even in the acellular pertussis vaccine era,^2,3^ these events are rare and post-vaccination fever in infants is generally self-limited. Post-vaccination fever can, however, lead to emergency room visits and other medical utilization, can result in unnecessary laboratory tests in febrile infants, and can cause a parent to miss time from work to care for a febrile child who cannot attend day care. Fever also results in discomfort for the child and can lead to disruption of sleep for both the parents and the child. Lastly, the occurrence of post-vaccination fever can influence parents’ perception of the safety of routine childhood immunizations.

Two randomized controlled trials conducted in the 1980s reported significant reductions in risk of post-vaccination fever in infants 2 through 6 months of age who received acetaminophen at the time of whole cell pertussis vaccination.^4,5^ In those trials, acetaminophen prophylaxis also led to significant reductions in the proportion of infants with fussiness and persistent crying in the day following vaccination. Prophylactic use of acetaminophen has not, however, been evaluated in infants receiving vaccines other than whole cell pertussis vaccine, and consequently there is no information regarding the magnitude or the benefit of prophylaxis, if present, in infants receiving the currently recommended complement of DTaP, *Haemophilus influenzae* b conjugate, hepatitis B, inactivated polio, and pneumococcal conjugate vaccinations.

We propose a randomized, controlled trial of acetaminophen prophylaxis among infants six weeks through nine months of age receiving routinely recommended vaccinations to address the following specific aims.

Primary Aim. To determine the efficacy of acetaminophen prophylaxis for prevention of post-vaccination fever (temperature ≥38ºC) following routine childhood immunizations.

Secondary Aims. To determine whether use of prophylactic acetaminophen reduces the infant’s medical utilization, parents’ time lost from work, and sleep lost by parents and infant within a day of vaccination.

# A.2. Role of CDC Investigator

Frank DeStefano, MD, MPH, with the Immunization Safety Office, Office of the Chief Science Officer, Centers for Disease Control and Prevention, is a co-investigator. He will provide input on the study design and analysis plan. He will review study findings and assist in the interpretation of those findings, and will participate in the writing of the manuscript reporting study findings.

# B. Background

# B.1. Fever following administration of pneumococcal conjugate and other currently recommended vaccinations

Information on the frequency of fever, defined as a rectal temperature ≥38ºC, in children receiving vaccinations currently recommended at 2, 4, and 6 months of age is available from the reports of two clinical trials. Table 1 reports safety results from the phase III trial of pneumococcal conjugate vaccine conducted at Northern California Kaiser.^6^ In that study, children received pneumococcal conjugate vaccine concomitantly with DTaP and other recommended vaccinations. Information from the Principal Investigator of that study indicates that the majority of children in the study received acetaminophen prophylaxis prior to vaccination (Black S, personal communication), and so the frequency of fever in the study population may not be reflective of the baseline frequency of post-vaccination fever in the absence of prophylaxis.

Table 1. Proportion of infants with fever within 48 hours of vaccination with pneumococcal conjugate, DTaP, and other routinely recommended vaccines, as reported by a large randomized controlled trial of pneumococcal conjugate vaccine.^6^

| Age | Maximum rectal temperature | |
| --- | --- | --- |
|  | ≥38ºC | ≥39ºC |
| 2 mo | 15.1% | 0.9% |
| 4 mo | 23.9% | 2.5% |
| 6 mo | 19.1% | 1.7% |

Table 2 reports the results of a trial of a hexavalent combination vaccine (DTaP-HepB-IPV-Hib) administered with pneumococcal conjugate vaccine at 2, 3, and 4 months in Germany.^1^

Table 2. Proportion of infants with fever within 4 days of vaccination with a hexavalent combination vaccine and pneumococcal conjugate vaccine.

.

| Age | Maximum rectal temperature | |
| --- | --- | --- |
|  | ≥38ºC | >39ºC |
| 2 mo | 42.8% | 4.6% |
| 3 mo | 49.3% | 4.1% |
| 4 mo | 33.5% | 2.4% |

These two trials indicate that temperature ≥38ºC is common following administration of pneumococcal conjugate vaccine and other currently recommended vaccine antigens in children ≤6 months of age.

**B.2. Trials of acetaminophen prophylaxis prior to whole cell pertussis vaccination**

In the 1980s, two randomized trials of acetaminophen in infants receiving whole cell pertussis vaccine documented the efficacy of prophylaxis in prevention of post-vaccination fever. The trial by Ipp et al^5^ included children receiving a combination DTwP-IPV vaccine and the trial by Lewis et al^4^ assessed children receiving DTwP vaccine. The sample sizes and designs of those two studies are summarized in Table 3. Both studies evaluated the use of multiple doses of acetaminophen for prophylaxis.

Table 3. Design of two randomized controlled trials of acetaminophen prophylaxis prior to whole cell pertussis vaccination.

|  | Ipp et al^5^ | Lewis et al^4^ |
| --- | --- | --- |
| Study population | 2-6 months, n=449  18 months, n=70 | 2-6 months, n=222  18 months, n=41  4-6 years, n=19 |
| Dose and schedule | 15 mg/kg given at the time of vaccination and at 4 and 8 hours post-vaccination while the child was awake | 40 mg for 2 month olds, 80 mg for 4-6 month olds, 120 mg for 18 month olds, and 240 mg for 4-6 year olds, given zero to 30 minutes before vaccination and at 3, 7, 12, and 24 hours after vaccination |
| Assessment of temperature | Rectal temp at 4, 8, 12, and 24 hours while awake | Temperature (method not specified) at 3, 7, 12, and 24 hours after vaccination |

Results of the two studies in children two through six months of age are reported in Table 4.

Table 4. Results of two randomized controlled trials of acetaminophen prophylaxis prior to whole cell pertussis vaccination.

| Outcome | Ipp et al  N=449 | | | | Lewis et al  N=222 | | |
| --- | --- | --- | --- | --- | --- | --- | --- |
|  | Placebo, % | Aceta-minophen, % | | RR  (95% CI) | Placebo, % | Aceta-minophen, % | RR  (95% CI) |
| Temp ≥38ºC | 43 | 27 | | **0.61**  **(0.47-0.81)** | 53 | 30 | **0.57**  **(0.41-0.79)** |
| Temp ≥38.5ºC | Not reported | | | | 19 | 11 | 0.60  (0.32-1.15) |
| Temp ≥39ºC | 13 | | 3 | **0.26**  **(0.11-0.58)** | Not reported | | |
| Fussiness/  Fretfulness | 59 | | 35 | **0.59**  **(0.48-0.73)** | 72 | 46 | **0.64**  **(0.51-0.81)** |
| Anorexia | 14 | | 7 | **0.51**  **(0.28-0.93)** | 25 | 17 | 0.69  (0.41-1.15) |
| Vomiting | 6 | | 5 | 0.96  (0.42-2.16) | 8 | 10 | 1.40  (0.59-3.28) |
| Persistent crying | 30 | | 18 | **0.60**  **(0.42-0.86)** | 15 | 8 | 0.52  (0.24-1.13) |
| Pain in vaccinated limb | 79 | | 71 | 0.90  (0.80-1.01) | 67 | 55 | 0.81  (0.66-1.01) |

The results of the two trials of acetaminophen prophylaxis prior to whole cell pertussis vaccination indicate that prophylaxis was effective in preventing post-vaccination fever and fussiness. These trials did not assess whether reduction in the risk of those outcomes was association with reduction in medical utilization or improvement of quality-of-life indicators for parents.

# B.3. Current recommendations for prophylaxis prior to childhood vaccinations

Current recommendations of both the American Academy of Pediatrics and the Advisory Committee on Immunization Practices (ACIP) note the option to give acetaminophen prophylaxis for childhood vaccinations, but neither promote nor discourage routine use of prophylaxis.

The statement from the American Academy of Pediatrics included in the 2003 Red Book is as follows.

Elective administration of acetaminophen or other appropriate antipyretic at the time of immunization with DTaP and at 4 and 8 hours after immunization may decrease the subsequent incidence of fever and local reactions.

The statement from the 2002 ACIP General Recommendations on Immunization^7^ is as follows.

Acetaminophen has been used among children to reduce the discomfort and fever associated with vaccination.

# B.4. Significance of the current proposal

In deciding whether to recommend acetaminophen prophylaxis, physicians would ideally balance the risks of the medication use against the likely benefits for the child and parents. While the safety profile of acetaminophen in children is well established, any medication is associated with some risk of adverse events. In addition, medication administration involves some inconvenience for the parents and is associated with a cost, albeit small in this case. Factors on the benefit side of the prophylaxis equation include the magnitude of the benefit, if present, in preventing discomfort due to fever and reducing medical utilization. Perhaps as a consequence of the limited evidence regarding the current benefit of prophylaxis, the language regarding prophylaxis in national immunization guidelines is deliberately non-directive, and provides little guidance for physicians or parents.

Evidence regarding the magnitude of the benefit, if present, associated with routine acetaminophen prophylaxis is therefore needed. If prophylaxis confers a benefit for infants receiving currently recommended immunizations, accurate information regarding the magnitude and type of benefit provided would allow parents, providers, and advisory groups to make evidence-based decisions regarding the use of prophylaxis. This would likely lead to greater acceptance of this intervention and more consistent use by parents. Conversely, if prophylaxis is not associated with a significant benefit, unnecessary doses of medications in infants should be avoided.

# C. Research Design and Methods

# C.1. Study design

Randomized, controlled trial of acetaminophen prophylaxis.

# C.2. Study population

Children six weeks through nine months of age receiving childhood immunizations at Group Health Cooperative.

# C.3. Inclusion/exclusion criteria

To ensure a relatively homogeneous study population, and to avoid possible concern regarding acetaminophen administration to very small babies, infants with a birthweight of <2500 grams or gestational age of <36 weeks will be excluded from enrollment prior to four months of age. Children who will be four months of age and older at the time of the vaccination visit may be enrolled, regardless of their birthweight or gestational age.

Children will be randomized to receive study medication only for vaccination visits that are expected to include two or more vaccine injections. If, at the time of recruitment, a child is due to receive, for example, only a hepatitis B vaccination at their next vaccination visit, they will not be randomized to receive study medication at that visit. If they are expected to have a vaccination visit with administration of two or more vaccinations before ten months of age, they are eligible for randomization to study drug for that visit.

In summary:

Inclusion criteria for the study are:

1. Child is a current Group Health enrollee.
2. Child will be seen at a Group Health clinic for a Well Child visit that is expected to include 2 or more vaccines after 6 wks and before 10 months of age.

Exclusion criteria for the study are:

1. If child was born at less than 36 weeks of gestation, the child is not eligible until 4 months of age or older.
2. If the child’s birth weight was less than 5.5 pounds (2500 grams), the child is not eligible until 4 months of age or older.

# C.4. Recruitment and enrollment

This trial will evaluate children six weeks through nine months of age receiving childhood vaccinations at Group Health. To recruit and enroll this group, potentially eligible children will be identified from the Group Health data systems on an ongoing basis during the study recruitment period.

Parents of those potentially eligible children will be mailed a letter providing information on the study and inviting them to return a postage-paid, trifold response card if they are interested in more information. On that response card, they will indicate their name, phone number, and times that they would prefer to be contacted. A study staff member will then contact the parent by phone to explain the study in more detail and answer questions. If the child is eligible and the parent is interested in participating, they will be mailed a consent form to sign and return. Study staff may also call parents who have received the invitation letter, but have not yet initiated contact, in order to inform them of the study and assess their interest in participating.

After returning the signed consent form, the parent will then be mailed an enrollment package including the study diary, study medication, study medication dosing instructions, digital thermometer, study staff contact information, and other informational materials. Study staff will follow-up with a phone call prior to the scheduled vaccination visit in order to clarify medication administration instructions, review the study diary, remind parents of the study procedures, and answer questions.

A PowerPoint presentation will be made available on the Center for Health Studies web page as a resource to help parents properly complete the study diary.

Children may participate in the study only once.

Parents who do not respond to the first invitation mailing may be sent a second invitation letter, approximately two months after the first.

Parents will receive a $25 gift card to Target as an incentive for participating in the study.

# C.5. Randomization

Participants will be randomized with equal probability to receive acetaminophen or placebo. To ensure balanced allocation during the enrollment period, the randomization schedule will be generated with a variable block size of between six and twelve. Participants will be randomized, and assigned a study number, after informed consent has been obtained from the parent.

# C.6. Study drug

**C.6.a. Dosage and timing of doses.** Children will receive a maximum of five doses of the study medication and all doses will be administered within 24 hours of vaccination. Parents will be encouraged to take the bottle of study medication to the vaccination visit, and to give the first dose at that visit, after the child’s weight has been assessed and within an hour before or after vaccination. If the parent is unable to give the first dose within an hour of vaccination, they will be asked to give the first dose as close as possible to the vaccination, and within the allowable window of four hours before through up to 24 hours after vaccination.

Parents will be instructed to give subsequent doses a minimum of four hours apart and to give the last dose of study medication no later than 24 hours after vaccination. Following the first dose, each subsequent dose should be given no earlier than, but as close to, 4 hours following the previous dose as possible. If the child is asleep, or is not available (e.g., is at daycare), at four hours after a previous dose, the subsequent dose may be given at an interval greater than four hours after the previous dose. A maximum of five doses of study medication should be given, and the last dose must be given no later than 24 hours after the study vaccination. There will thus be variability in the intervals between consecutive doses of study medication, and in the total number of doses administered within the 24-hour period. The minimum interval between dose one and dose two, for example, is four hours, and the maximum interval between those two doses is 24 hours, in which case the child would receive a total of only two doses in the study period. If all doses are given exactly on schedule, doses would be given at 0, 4, 8, 12, and 16 hours after vaccination.

Table 5. Study drug dosing schedule.

|  | Recommended timing | Allowable interval |
| --- | --- | --- |
| Dose 1 | ±1 hour of vaccination | Within 4 hours before or up to 24 hours following vaccination |
| Dose 2 | 4 hours following dose 1 | No earlier than 4 hours following dose 1, and no later than 24 hours following vaccination |
| Dose 3 | 4 hours following dose 2 | No earlier than 4 hours following dose 2, and no later than 24 hours following vaccination |
| Dose 4 | 4 hours following dose 3 | No earlier than 4 hours following dose 3, and no later than 24 hours following vaccination |
| Dose 5 | 4 hours following dose 4 | No earlier than 4 hours following dose 4, and no later than 24 hours following vaccination |

**C.6.b. Study drug formulation and dispensement.** Study drug and placebo will be formulated as liquid suspensions, flavored to mask differences in taste, and packaged in identical containers by the study pharmacy. Study drug will be dispensed to parents by mail after they have signed and returned informed consent for study participation and prior to their vaccination appointment.

Study drug will be formulated to provide 160 mg of acetaminophen per 5 cc dose. Dosages of acetaminophen will be based on weight, as assessed at the vaccination visit, to provide a dose of between 10 and 15 mg of acetaminophen per kilogram of body weight. The parents will be provided with a simplified version of the information on dose volume to be administered per weight in pounds. The study drug package will include a 5.0 cc medication syringe graduated in 0.5 cc intervals.

(Note: a weight of 7 pounds is below the 5^th^ percentile for 6 week old girls, and a weight of 26 pounds is above the 95^th^ percentile for 10 month old boys.)

Table 6. Dosage table for acetaminophen solution (160 mg/5 cc dose) by weight in pounds.

| Weight in pounds | Weight in kg | 10 mg/kg dose | 15 mg/kg dose | Dose volume in 0.5 cc increments | Mg per dose volume | Mg/kg per dose volume |
| --- | --- | --- | --- | --- | --- | --- |
| 7 | 3.2 | 32 | 48 | 1.5 | 48 | 15.0 |
| 8 | 3.6 | 36 | 54 | 1.5 | 48 | 13.3 |
| 9 | 4.1 | 41 | 61 | 1.5 | 48 | 11.7 |
| 10 | 4.6 | 46 | 69 | 2.0 | 64 | 13.9 |
| 11 | 5.0 | 50 | 75 | 2.0 | 64 | 12.8 |
| 12 | 5.4 | 54 | 81 | 2.5 | 80 | 14.8 |
| 13 | 5.9 | 59 | 88 | 2.5 | 80 | 13.5 |
| 14 | 6.4 | 64 | 96 | 3.0 | 96 | 15.0 |
| 15 | 6.8 | 68 | 102 | 3.0 | 96 | 14.1 |
| 16 | 7.3 | 73 | 109 | 3.0 | 96 | 13.1 |
| 17 | 7.7 | 77 | 115 | 3.5 | 112 | 14.5 |
| 18 | 8.2 | 82 | 123 | 3.5 | 112 | 13.6 |
| 19 | 8.6 | 86 | 129 | 4.0 | 128 | 14.9 |
| 20 | 9.1 | 91 | 136 | 4.0 | 128 | 14.1 |
| 21 | 9.5 | 95 | 142 | 4.0 | 128 | 13.5 |
| 22 | 10.0 | 100 | 150 | 4.5 | 144 | 14.4 |
| 23 | 10.4 | 104 | 156 | 4.5 | 144 | 13.8 |
| 24 | 10.9 | 109 | 163 | 5.0 | 160 | 14.7 |
| 25 | 11.4 | 114 | 171 | 5.0 | 160 | 14.0 |
| 26 | 11.8 | 118 | 177 | 5.0 | 160 | 13.6 |

If a child is expected to be below the minimum weight on the dosing schedule at their next vaccination visit, their participation in the study would be delayed until a vaccination visit when they were at or above the minimum weight. If a child is above the maximum weight listed in the table, they will receive the maximum dosage listed on the table.

# C.7. Provisions for unblinding

Study staff will maintain the capacity to unblind the randomization assignment on a 24/7 basis. If the parent or a health care provider has a concern regarding a possible adverse event to the study medication, the medication may be stopped, and, if needed, the randomization assignment unblinded.

If the parent or a health care provider believes the child needs acetaminophen treatment for any reason, the parent or health care provider may call study staff at any time to request unblinding of the randomization assignment.

Each participant’s randomization assignment will be stored in an individual sealed, opaque envelope labeled with the corresponding study number. Study staff will have access to the envelopes for children who have been randomized but have not completed study drug therapy. If the need for unblinding arises, study staff will open the envelope corresponding to the number of the infant for whom unblinding is requested. Thus, the unblinding process will unblind the study staff only for that individual child, and not for other participants. The study number will be provided on the medication bottle dispensed to parents.

After unblinding, study staff will refer the parent to the consulting nurse or the child’s physician for further clinical management, if needed, of the febrile illness or other symptoms that led to the request for acetaminophen treatment.

# C.8. Outcomes

The primary outcome is fever, defined as a rectal temperature ≥38ºC, within 32 hours of vaccination. This time interval was chosen because it corresponds to 8 hours after the latest possible administration of the last dose of study medication (to be given no later than 24 hours after vaccination) and thus encompasses the maximum window of expected activity of the study medication.

Secondary outcomes include the following.

1. High fever, defined as rectal temperature ≥39ºC, within 32 hours of vaccination.
2. Medical utilization. Parents will be asked to report telephone calls to the consulting nurse or their physician’s office that were made due to concerns regarding an acute illness, fever, or possible vaccine reaction, on the day of or the day following vaccination. They will also be asked to report outpatient, urgent care, and emergency room visits on the day of or the day following immunization that were for evaluation of an acute illness (i.e., not scheduled prior to the vaccination visit), fever, or a possible vaccine reaction.

Several categories of medical utilization will be evaluated, including any utilization within 32 hours of vaccination, a telephone consult or outpatient clinic visit within that period, or an urgent care or emergency room visit within that period.

1. Parent time lost from work to care for infant. Parents will be asked to report whether they were scheduled to work on the day of or the day following vaccination and, if so, whether they had to miss work to care for their infant on those days. They will be asked not to report work missed to receive immunizations.
2. Time lost from sleep. Parents will be asked about their sleep and the child’s sleep on the night following vaccination. Each parent will be asked to indicate whether they slept much less than usual, less than usual, about the usual­ amount, more than usual, or much more than usual on that night. They will also be asked whether the baby slept much less than usual, less than usual, about the usual­ amount, more than usual, or much more than usual on that night.
3. Infant fussiness. In the study diary, parents will be asked to record level of fussiness (compared with the child’s usual) within 32 hours of vaccination.
4. Unblinding of study drug assignment. The need for unblinding, including the timing of and the precipitating reason, will be assessed.

# C.9. Data collection

Parents will complete a study diary and return it by mail, using a postage paid envelope provided. They will be asked to record the child’s weight assessed at the vaccination visit and the time that vaccinations were administered. In the 24 hours after vaccination, they will be asked to record the timing and dosage of each dose of study medication administered. If they discontinue the study medication, they will be asked the reasons for that action.

The primary outcome of fever will be assessed based on rectal temperature measurements recorded by the parent on the day of and the day following vaccination. Parents will be asked to assess and record rectal temperature at the time that study medication doses 2 though 5 are given. If they are unable to assess rectal temperature, the study medication dose may be given without concurrent assessment of temperature. A final rectal temperature measurement should be obtained approximately 24 hours after vaccination, or four hours after the final dose of study medication, whichever is later. At each study medication dosage time, and at the time of the final temperature assessment, parents will also record the child’s level of fussiness (compared with the child’s usual level).

On the morning following vaccination, parents will also record the relative amount of sleep that each parent and the child had on the night of vaccination. Parents will be asked to report whether they were scheduled to work on the day of or the day following the child’s vaccination visit and, if so, whether either parent missed work to care for the child due to fever, fussiness, or possible vaccine reaction.

Parents will be asked to record any use of medical services for fever or other acute symptoms following the vaccination visit through the next day. This would include telephone calls to the physician office or the consulting nurse service, unscheduled medical outpatient visits, and emergency room and urgent care visits. Parents will not be asked to report routine, previously scheduled visits. If the child is admitted to the hospital on the day of or the day following vaccination, this information will be collected.

If the parent elects to stop the study medication, they will be asked to record a final temperature and fussiness at 24 hours post-vaccination, or four hours after the last dose of study medication, whichever is later. Parents will also be asked to record information on sleep, time lost from work, and medical utilization on the day of or the day following vaccination, in the same way that they would have if the study medication had been continued.

Prior to returning the study diary, parents will be also asked to guess whether their child received acetaminophen or placebo, to judge the adequacy of the blinding procedure.

Information on injection site reactions will not be specifically collected. These reactions are generally mild in infants and are unlikely to lead to medical care or to parents’ time lost from work. Collection of data on local reactions would involve a relatively complex data collection system, as children may receive up to five immunizations at a single visit. If a parent seeks medical attention for an acute illness or problem on the day of or the day following vaccination, however, they will be asked to record that utilization and to identify the reason for the contact.

Vaccinations given at the vaccination visit will be identified from Group Health immunization records.

# C.10. Power calculations and sample size

Assuming that 40% of children in the placebo group will meet the primary outcome definition, and that children will be randomized equally to acetaminophen or placebo, evaluation of 897 children would allow 80% power to detect a 30% reduction in risk of the primary outcome associated with treatment. Assuming that study diary information will not be available for 10% of children randomized, we estimate that randomization of 1000 children would give an adequate number of evaluable participants (with study diary information) to achieve 80% power to detect a 30% reduction in risk of the primary outcome.

Each month, there are approximately 875 children at Group Health who are scheduled to receive immunizations recommended at 2, 4, and 6 months of age. We expect enrollment to be completed in 24-36 months.

# C.11. Statistical analysis

The primary analysis will be an intent-to-treat analysis and will include all evaluable participants with study diary information. A secondary per protocol analysis will also be performed, which will be restricted to the subgroup of evaluable participants with study drug administration meeting pre-specified criteria. Compliance per protocol will be defined as administration of the first dose of the study medication within four hours before through one hour after vaccination, and administration of at least two additional doses, given at least 4 hours apart, within 24 hours of vaccination.

The proportion of participants with each outcome (i.e., with fever, high fever, any medical utilization, any parent work loss, any parent/infant sleep loss, any fussiness beyond usual levels, and required unblinding) will be compared between treatment groups using a chi-square test of independence. To examine the impact of possible differences between treatment groups, we will also compare outcome proportions that are adjusted for other factors. Specifically, we will model the probability of each binary outcome as an exponential function of risk factors and perform nonlinear least squares estimation to obtain asymptotically unbiased estimates of the relative risk of the outcome for placebo versus treatment. To account for misspecification of the variance, we will compute model-robust (Huber-White) standard errors.

# D. Efficacy and safety monitoring

Both efficacy and safety will be monitored regularly throughout the study. Results of interim monitoring will be used to alert investigators about potential early beneficial or harmful treatment effects and to inform decisions about early study termination. This information, however, will not be used as the sole basis for stopping the study. Results of interim analyses will be reported first in a closed session to the medical monitor and then in an open session to the monitor along with the investigators.

**D.1. Efficacy monitoring**

To monitor the trial for efficacy, during the study the biostatistician will perform two interim analyses of the primary study outcome (rectal temperature ≥38ºC), after enrollment of 333 and 666 participants. This level of monitoring should be frequent enough to detect any early beneficial effects but infrequent enough to allow an adequate amount of new data to accumulate for analysis. Enrollment will continue during the interim analyses. To account for testing at multiple interim time points and thus maintain the desired overall type 1 error rate of incorrectly rejecting the null hypothesis of no treatment effect at the standard 0.05 level, we will use the group sequential spending function methodology developed by Lan and DeMets.^8^ Specifically, we will use the O’Brien and Fleming alpha-spending function, α(t_i_*)= 2 - 2Φ(Z_(α/2)_/t_i_*), where t_i_* is the expected proportion of participants available at the i^th^ interim analysis, Φ is the cumulative normal distribution function, and Z_(α/2)_ is the normal critical value that corresponds to a rejection probability of alpha. Assuming a one-sided test, the p-values required to reject the null hypothesis of no treatment effect at each interim analysis and at the final analysis are 0.0007, 0.0164, and 0.0499, respectively. Depending on the results of the first interim analysis, we may adapt the number and timing of additional interim analyses if more or less monitoring is determined to be needed.

# D.2. Safety monitoring

The safety profile of acetaminophen in children is well established, and adverse events are rare in children given recommended dosages of acetaminophen. There are therefore no adverse events that are expected as a consequence of administration of the acetaminophen dosages specified for this study.

The trial will, however, include monitoring for possible unexpected adverse events. In this trial, three categories of safety outcomes will be defined. These categories will include 1) evidence of misuse of the study drug, 2) concomitant administration of other acetaminophen-containing medications, and 3) the occurrence of severe febrile outcomes.

Events meeting the definition of misuse of the study drug or concomitant administration of other acetaminophen-containing medications will be reported to the medical monitor as they occur, in order to allow changes to the study protocol or procedures to be implemented if required. Severe febrile outcomes will also be reported to the medical monitor as they occur, and in addition will be formally monitored and tested by the study biostatistician in the same manner as described for the primary efficacy outcome monitoring.

**D.2.a. Safety outcomes.** The following safety outcomes will be identified.

1. Misuse of the study drug. Parents will be instructed to report, on the study diary, the time of administration of each dose of study drug and the volume of study drug given for each dose. Misuse of study drug will be defined by administration of a greater than recommended dosage, administration of consecutive doses at less than the minimum interval between doses, and administration of more than the maximum number of doses. Specifically, misuse will be defined by any one of the following, including a) report of administration of a dose of study medication that is >2 times the recommended weight-based dose, b) administration of consecutive doses within an interval of three hours or less, or c) administration of a total of more than five doses of study drug.

Any report of misuse of the study drug will trigger further follow-up with the parent. Unless there was clearly an error in recording or transcription of the study drug administration information, any of the above patterns of study drug use will be classified as misuse of the study drug. Misuse events will be reported to the PI and to the medical monitor within five working days of their initial identification by study staff. The medical monitor, with consultation with the investigators or other individuals as appropriate, will determine if changes in the study protocol or study procedures are required.

If study staff identify a pattern of administration that, in their clinical judgment, could constitute a clinically significant overdose of acetaminophen, if the study medication contained acetaminophen, they will immediately consult with the PI and will unblind the randomization assignment. If the child had been randomized to acetaminophen, study staff and the PI will consult with the medical monitor and appropriate clinical personnel.

1. Concomitant administration of other acetaminophen-containing medications. Parents will be asked to record all medications, both over-the-counter and prescription, given on the day of or the day following vaccination. Informational materials given to parents before the vaccination day will include instructions not to give the child over-the-counter or prescription medications that contain acetaminophen on the day of vaccination or within 16 hours of the last dose of study medication. The informational materials will include a list of generic and trade names of common medications that contain acetaminophen. If a parent reports administration of an acetaminophen-containing product on the day of vaccination or within 15 hours of the last dose of study medication, this will be classified as a concomitant administration safety outcome. These events will prompt further follow-up with the parents and, unless clearly due to a recording error, will be reported to the principal investigator and the medical monitor within five working days of initial identification by study staff. The medical monitor, with consultation with the investigators or other individuals as appropriate, will determine if changes in the study protocol or study procedures are required.
2. Severe fever events. To determine if relatively severe febrile outcomes are occurring at an unacceptable frequency in the placebo group, the outcomes of reported temperature ≥39.5ºC, hospitalization for a febrile illness, and febrile seizures (regardless of hospitalization status) that occur on the day of or the day following vaccination will be identified from information reported on the study diary. Each of these events will be reported to the principal investigator and the medical monitor within five working days of initial identification by study staff. A summary will also be maintained by the study biostatistician, who, at the time of each interim analysis, will report frequencies of the severe fever events by unblinded study group first to the medical monitor in a closed session and then to the investigators. The medical monitor and investigators, in consultation with other experts as appropriate, will determine whether there are safety concerns that warrant consideration of changing the study protocol or procedures or stopping the trial for safety.

Reference List

1. Tichmann-Schumann I, Soemantri P, Behre U, Disselhoff J, Mahler H, Maechler G, et al. Immunogenicity and reactogenicity of four doses of diphtheria-tetanus-three-component acellular pertussis-hepatitis B-inactivated polio virus-Haemophilus influenzae type b vaccine coadministered with 7-valent pneumococcal conjugate Vaccine. Pediatr Infect Dis J 2005; 24:70-7.

2. Jackson LA, Carste BA, Malais D, Froeschle J. Retrospective population-based assessment of medically attended injection site reactions, seizures, allergic responses and febrile episodes after acellular pertussis vaccine combined with diphtheria and tetanus toxoids. Pediatr Infect Dis J 2002; 21:781-6.

3. Wise RP, Iskander J, Pratt RD, Campbell S, Ball R, Pless RP, et al. Postlicensure safety surveillance for 7-valent pneumococcal conjugate vaccine. JAMA 2004; 292:1702-10.

4. Lewis K, Cherry JD, Sachs MH, Woo DB, Hamilton RC, Tarle JM, et al. The effect of prophylactic acetaminophen administration on reactions to DTP vaccination. Am J Dis Child 1988; 142:62-5.

5. Ipp MM, Gold R, Greenberg S, Goldbach M, Kupfert BB, Lloyd DD, et al. Acetaminophen prophylaxis of adverse reactions following vaccination of infants with diphtheria-pertussis-tetanus toxoids-polio vaccine. Pediatr Infect Dis J 1987; 6:721-5.

6. Black S, Shinefield H, Fireman B, Lewis E, Ray P, Hansen JR, et al. Efficacy, safety and immunogenicity of heptavalent pneumococcal conjugate vaccine in children. Northern California Kaiser Permanente Vaccine Study Center Group. Pediatr Infect Dis J 2000; 19:187-95.

7. Atkinson WL, Pickering LK, Schwartz B, Weniger BG, Iskander JK, Watson JC. General recommendations on immunization. Recommendations of the Advisory Committee on Immunization Practices (ACIP) and the American Academy of Family Physicians (AAFP). MMWR Recomm Rep 2002; 51:1-35.

8. DeMets DL, Lan KK. Interim analysis: the alpha spending function approach. Stat Med 1994; 13:1341-52; discussion 1353-6.
